# Peripheral Exophytic Oral Lesions: A Clinical Decision Tree

**DOI:** 10.1155/2017/9193831

**Published:** 2017-07-05

**Authors:** Hamed Mortazavi, Yaser Safi, Maryam Baharvand, Somayeh Rahmani, Soudeh Jafari

**Affiliations:** ^1^Department of Oral Medicine, School of Dentistry, Shahid Beheshti University of Medical Sciences, Tehran, Iran; ^2^Department of Oral and Maxillofacial Radiology, School of Dentistry, Shahid Beheshti University of Medical Sciences, Tehran, Iran

## Abstract

Diagnosis of peripheral oral exophytic lesions might be quite challenging. This review article aimed to introduce a decision tree for oral exophytic lesions according to their clinical features. General search engines and specialized databases including PubMed, PubMed Central, Medline Plus, EBSCO, Science Direct, Scopus, Embase, and authenticated textbooks were used to find relevant topics by means of keywords such as “oral soft tissue lesion,” “oral tumor like lesion,” “oral mucosal enlargement,” and “oral exophytic lesion.” Related English-language articles published since 1988 to 2016 in both medical and dental journals were appraised. Upon compilation of data, peripheral oral exophytic lesions were categorized into two major groups according to their surface texture: smooth (mesenchymal or nonsquamous epithelium-originated) and rough (squamous epithelium-originated). Lesions with smooth surface were also categorized into three subgroups according to their general frequency: reactive hyperplastic lesions/inflammatory hyperplasia, salivary gland lesions (nonneoplastic and neoplastic), and mesenchymal lesions (benign and malignant neoplasms). In addition, lesions with rough surface were summarized in six more common lesions. In total, 29 entities were organized in the form of a decision tree in order to help clinicians establish a logical diagnosis by a stepwise progression method.

## 1. Introduction

Lesions in the oral cavity generally present as ulcerations, red-white lesions, pigmentations, and exophytic lesions. Clinical classification of oral lesions is of great importance in the diagnostic process [1, 2]. The term oral exophytic lesions is described as pathologic growths projecting above the normal contours of the oral mucosa [2]. There are several underlying mechanisms responsible for oral exophytic lesions such as hypertrophy, hyperplasia, neoplasia, and pooling of the fluid [1], which makes it difficult to approach such lesions clinically [3, 4]. According to a national epidemiologic study by Zain et al., exophytic lesions account for 26% of all oral lesions [3]. Therefore, attempts should be done to arrive at a timely diagnosis via more logical routes like decision trees rather than test-and-error methods [3, 4]. Exophytic lesions can be classified according to their surface texture (smooth and rough), type of base (pedunculated, sessile, nodular, and dome shape), and consistency (soft, cheesy, rubbery, firm, and bony hard) [1, 4]. This narrative review paper, however, focuses on the surface shapes of the lesions as the main clinical feature in order to build a diagnostic decision tree. In this regard, oral peripheral exophytic lesions are classified as lesions with rough surface and those with smoothly contoured shape [1, 5, 6].

## 2. Methodology

General search engines and specialized databases including PubMed, PubMed Central, Medline Plus, EBSCO, Science Direct, Scopus, Embase, and authenticated textbooks were used by the first author and the corresponding author to find relevant topics by means of MeSH keywords such as “oral soft tissue lesion,” “oral tumor like lesion,” “oral mucosal enlargement,” and “oral exophytic lesion.” Related English-language articles published since 1988 to 2016 in both medical and dental journals including reviews, meta-analyses, original papers (randomized or nonrandomized clinical trials; prospective or retrospective cohort studies), case reports, and case series on oral disease were appraised.

**Figure 1:**
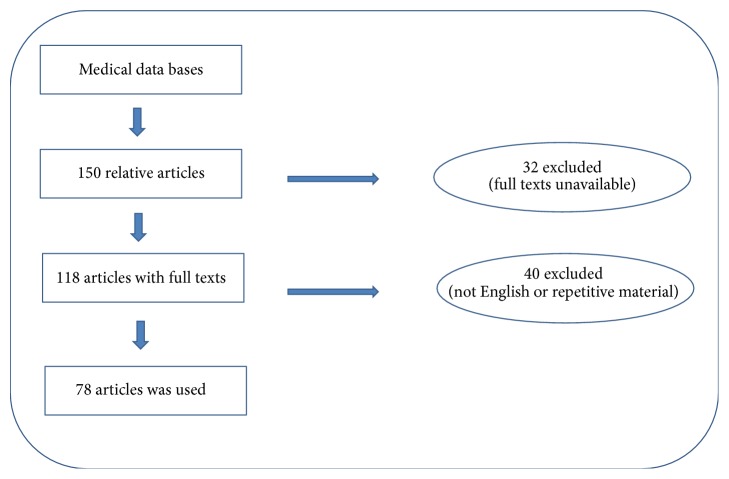
Flowchart for choosing eligible articles.

**Figure 2:**
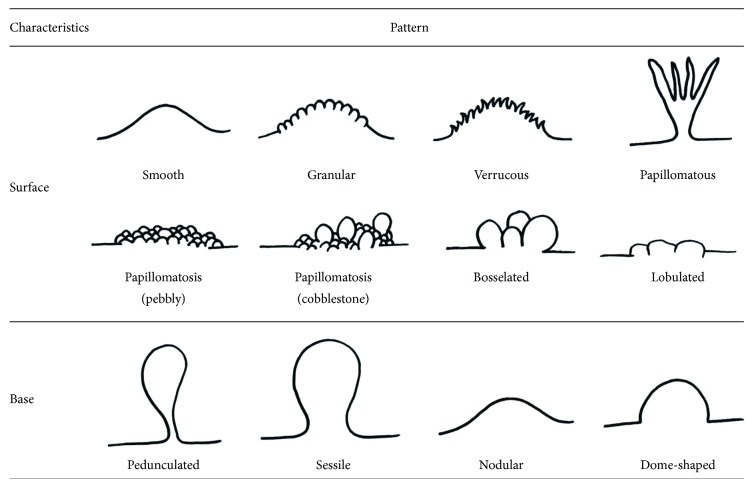
Schematic view of surface and base characteristics of oral exophytic lesions.

**Figure 3:**
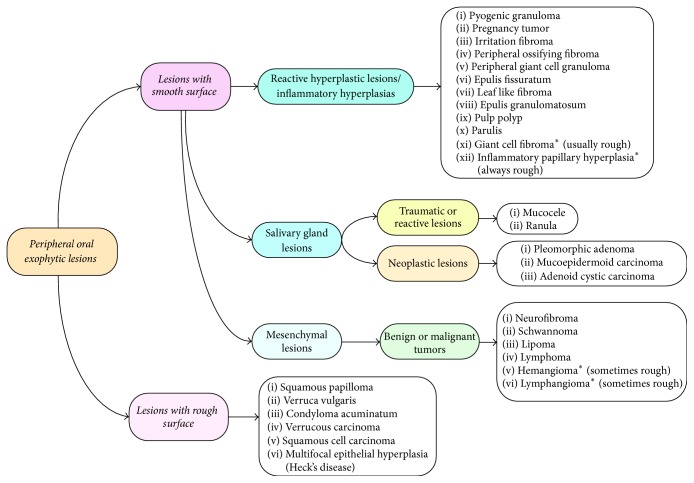
Decision tree for peripheral oral exophytic lesions.

Out of about 150 related articles, 72 were excluded due to lack of full texts, being written in languages other than English or containing repetitive material. Finally, three textbooks and 78 papers were selected including 13 reviews, 55 case reports or case series, and 10 original articles (Figure 1). In this article, peripheral oral exophytic lesions were categorized into two major groups according to their surface texture: smooth (mesenchymal or nonsquamous epithelium-originated) and rough (squamous epithelium-originated) (Figure 2). Lesions with smooth surface were also categorized into three subgroups according to their general frequency: reactive hyperplastic lesions/inflammatory hyperplasia, salivary gland lesions (nonneoplastic and neoplastic), and mesenchymal lesions (benign and malignant neoplasms). In addition, lesions with rough surface were summarized in six more common lesions. In total, 29 entities were organized in the form of a decision tree (Figure 3) in order to help clinicians establish a logical diagnosis by a stepwise progression method.

## 3. Lesions with Smooth Surface

### 3.1. Reactive Hyperplastic Lesions/Inflammatory Hyperplasias

Reactive hyperplasia is the most frequent phenomenon responsible for exophytic lesions in the oral cavity (Table 1). These lesions represent a reaction to some kind of chronic trauma or low grade injuries such as fractured tooth, calculus, chewing, and iatrogenic factors including overextended flange of dentures and overhanging dental restorations [7]. Reactive lesions are usually seen on the gingivae followed by the tongue, buccal mucosa, and floor of the mouth. Clinically, they appear as pedunculated or sessile masses with smooth surface. Lesions are varied from pink to red and soft to firm in terms of color and consistency [7, 8]. However, the clinical features resemble neoplastic lesions in some instances, which cause a diagnostic dilemma. The most common entities of reactive nature are pyogenic granuloma, pregnancy epulis, irritation fibroma, peripheral ossifying fibroma, peripheral giant cell granuloma, epulis fissuratum, leaf-like fibroma/fibroepithelial polyp, parulis, pulp polyp, epulis granulomatosum, giant cell fibroma, and inflammatory papillary hyperplasia/palatal papillomatosis [1, 4, 7].

#### 3.1.1. Pyogenic Granuloma

**Figure 4:**
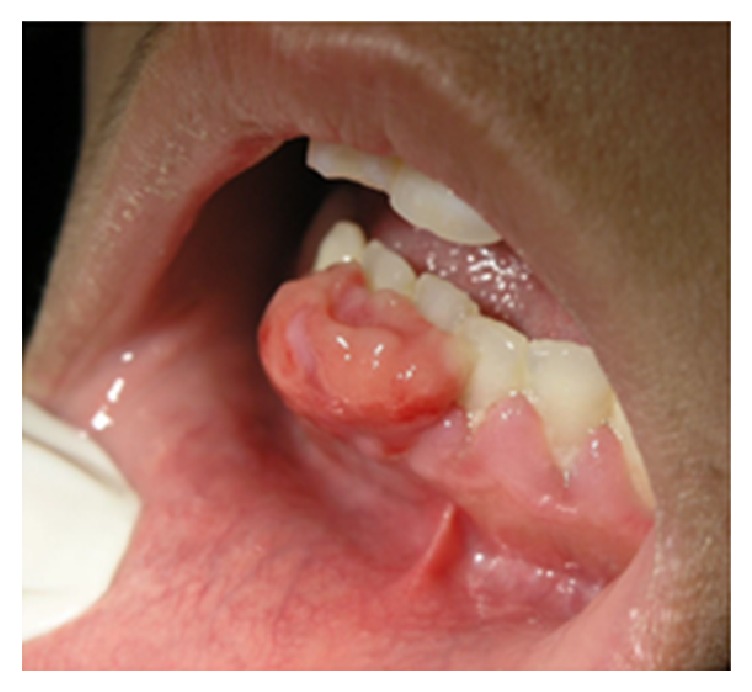
Pyogenic granuloma as a sessile lesion on mandibular labial gingivae with an ulcerated smooth surface.

Pyogenic granuloma is a common tumor-like lesion in the oral cavity appearing as a smooth or lobulated, asymptomatic mass that is usually pedunculated or sessile. The surface is characteristically ulcerated and friable, which may be covered by a yellow fibrinous membrane (Figure 4) [4, 9]. Depending on the duration of the lesion, its color ranges from shiny red to pink to purple with soft to firm in palpation. It often bleeds easily because of its extreme vascularity. Approximately in one-third of the lesions a history of trauma can be detected [10]. Pyogenic granuloma may exhibit a rapid growth, but it usually reaches its maximum size within weeks or months. The size of the lesion varies from a few millimeters to several centimeters in diameter. In 75% of all cases, the most frequently affected site is the gingivae followed by the lips, tongue, and buccal mucosa. Maxillary gingivae are more affected than mandibular gingivae, and anterior areas more frequently involved than posterior areas [9–11]. There is a female predilection and a tendency to affect children and young adults. Patients with pyogenic granuloma are treated by conservative surgical excision. However, recurrence is not uncommon [9].

#### 3.1.2. Pregnancy Tumor

Pregnancy tumor or granuloma gravidarum is a reactive lesion with the same clinical features to pyogenic granuloma. The lesion may emerge during the first trimester with a gradual increasing incidence to the seventh months of pregnancy, which is presumably related to the rising levels of estrogen and progesterone. Some of these lesions resolve spontaneously after delivery or undergo fibrous maturation mimicking an irritation fibroma [4, 10]. The lesions do not occur in people with optimum oral hygiene suggesting local irritation as an important etiologic factor [4]. Patients with small isolated lesions and otherwise healthy gingivae might be monitored for shrinkage after delivery, but large lesions or generalized pregnancy gingivitis or periodontitis warrants the need for treatment during pregnancy [4].

#### 3.1.3. Irritation Fibroma

**Figure 5:**
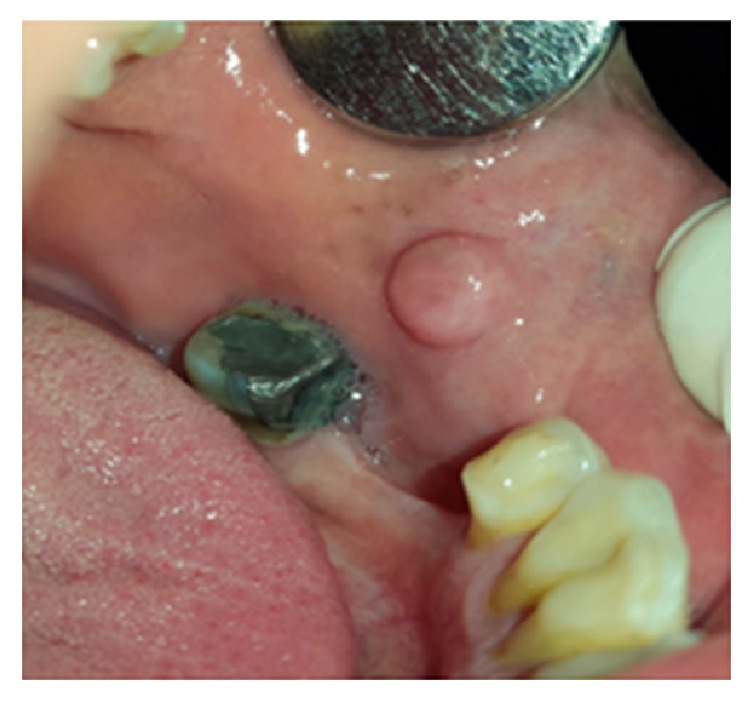
Irritation fibroma on the buccal mucosa with a smooth surface and dome-shaped base.

Irritation fibroma or focal fibrous hyperplasia is the most common tumor-like lesion of the oral cavity with the prevalence of 1-2% in general population. It appears as an asymptomatic, pedunculated, or sessile exophytic lesion with a smooth surface and being similar to the surrounding mucosa in color (Figure 5). However, the surface may show hyperkeratosis from secondary trauma. It can be firm and resilient or soft with spongy consistency. Although fibromas usually reach to 1.5 cm or less in diameter they might appear as very tiny to quite large lesions. The most commonly affected site is the buccal mucosa along the line of occlusion; however it can occur anywhere in the oral cavity. The labial mucosa, tongue, and gingivae can be involved as well. It is likely that many fibromas represent fibrous maturation of a preexisting pyogenic granuloma. There is a female predilection with female to male ratio of 2 : 1 with the majority of cases being reported in the fourth to sixth decades of life. Conservative surgical excision is the treatment of choice for irritation fibroma with a low recurrence rate.

#### 3.1.4. Peripheral Ossifying Fibroma

Peripheral ossifying fibroma also called peripheral fibroma with calcification, ossifying fibroid epulis, and calcifying fibroblastic granuloma is a reactive gingival enlargement [10]. It represents 2% to 9% of all gingival lesions and 3% of all oral lesion biopsy samples [12, 13]. The lesion appears as a smooth, pedunculated, or sessile, firm to hard mass that usually emanates from the interdental papilla. It is a red to pink lesion often less than 2 cm in diameter, but lesions up to 8 cm have been reported as well [10, 13]. Tooth mobility, tooth migration, and bone destruction have been noticed in some cases [13]. This lesion occurs exclusively on the gingivae with up to 60% of cases being reported in the anterior areas of the maxilla (incisor-cuspid region) [12, 13]. Two theories have been proposed to explain the pathogenesis of the lesion: it might originate from a calcified pyogenic granuloma, or it may arise from an overgrowth and proliferation of different components of connective tissue in the periodontium, but the main etiology is yet to be elucidated [12, 14]. Peripheral ossifying fibroma is predominantly a lesion of teenagers and young adults with a peak prevalence being between 10 to 19 years [10]. Females are more affected than males, mainly during their second decade of life, due to fluctuations of estrogen and progesterone [12]. Treatment usually involves surgical excision, and the lesion should be excised down to the periosteum. The recurrence rate has been estimated to be between 8% and 20% [10, 12].

#### 3.1.5. Peripheral Giant Cell Granuloma

**Figure 6:**
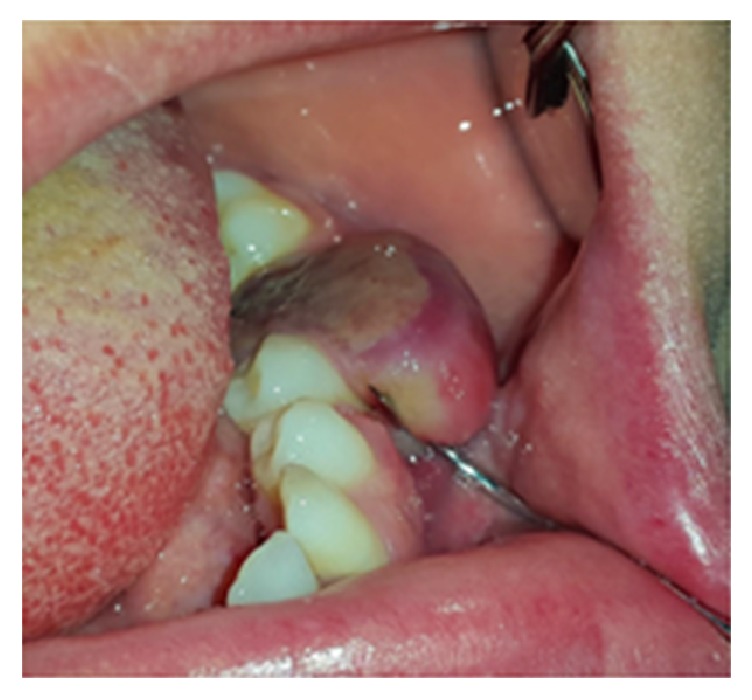
PGCG with an ulcerated, smooth surface and purplish color located buccolingually on the left mandibular ridge.

Peripheral giant cell granuloma is a common tumor-like lesion of the oral cavity that conveys a reactive response in the periodontium, periodontal ligament and gingivae. It occurs exclusively on the edentulous alveolar ridge and gingivae as a smooth, reddish-blue, pedunculated, sessile, or nodular mass, which is firm to palpation. In some cases the clinical appearance of the lesion is similar to pyogenic granuloma; however peripheral giant cell granuloma is more bluish-purple colored as compared with bright red color of pyogenic granuloma (Figure 6) [10, 15]. It is usually less than 2 cm in diameter, but larger sizes are seen occasionally. Progressive growth in some cases may lead to bone and root resorption [16]. The mandible is more affected than the maxilla, and there is a female predilection with female to male ratio of 2 : 1 [15, 16]. As the giant cells are found to act as a potential target for estrogen, it is not surprising that the lesions are triggered by sex hormones [17]. The lesions can develop at any age; however peak prevalence was found in the fifth and sixth decades of life [10]. Hyperparathyroidism should be considered in differential diagnosis in case of multiple lesions especially with a history of recurrences. In spite of adequate treatment, children with hypophosphatemic rickets are also at a higher risk for developing such lesions [16, 17]. Complete surgical excision with elimination of the entire base of the lesion is the accepted treatment plan for this lesion [15–17].

#### 3.1.6. Epulis Fissuratum

**Figure 7:**
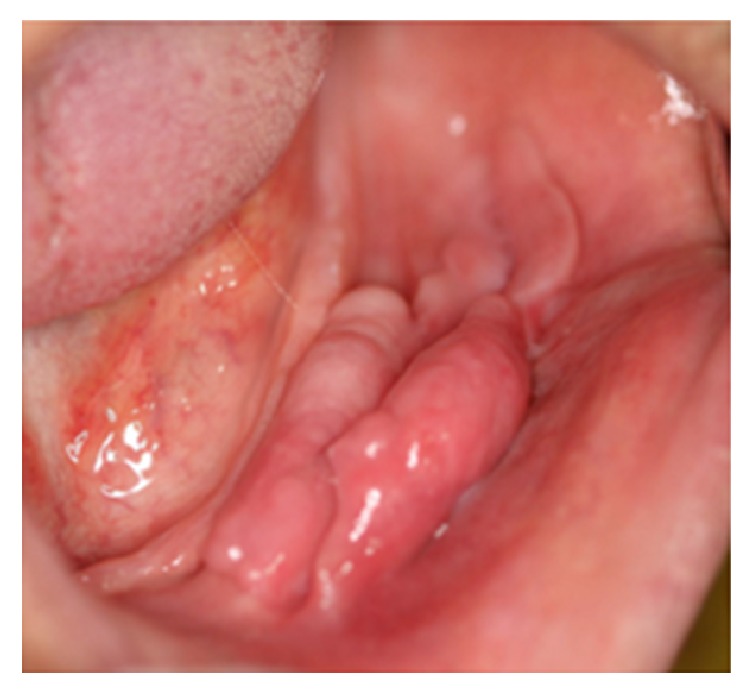
Massive epulis fissuratum presenting as an exophytic lesion with smooth surface associated with an ill-fit mandibular denture.

Epulis fissuratum or denture-induced hyperplasia is a reactive lesion of the oral cavity caused by low grade chronic trauma from dentures [18]. About 70% of patients wear ill-fit dentures continuously all day long for more than 10 years [19]. The lesion appears as an asymptomatic single fold or multiple folds of hyperplastic tissues in the alveolar vestibule along denture flanges with a smooth surface, soft to firm consistency, and a normal coloration (Figure 7) [18, 20]. In some cases, severe inflammation or ulceration may be seen in the bottom of the folds [18]. It has been reported in 5% to 10% of the jaws fitted with dentures and is more prevalent in the maxilla than the mandible, especially on the facial aspect of the alveolar ridges [18, 19]. The anterior portions of the jaws are more often affected; however epulis fissuratum of the soft palate has been reported in the literature [20]. Two-thirds to three-fourths of all cases have been found in females [19, 20]. In women, postmenopausal hormonal imbalance makes the oral mucosa susceptible to a hyperplastic growth [18]. It is more frequent in patients over 40 years; however this entity has been reported in patients from childhood to elderly [1]. The size of the lesion varies from less than 1 cm in diameter to massive lesions involving extensive areas on the vestibule [19, 20]. Epulis fissuratum can be treated conservatively or surgically depending on the size of the lesion.

#### 3.1.7. Leaf-Like Fibroma

Ill-fit dentures worn for many years can cause benign hyperplastic fibrous growths or epulides. When a fibrous epulis forms underneath the palatal base of a denture, it is known as a leaf-like denture fibroma or fibroepithelial polyp [21]. It is characterized as a pain less, flattened, pink, firm mass attached to the palate by a narrow stalk or a broad base (pedunculated or sessile) which can reach to several centimeters in diameter. The edge of the lesion is serrated resembling a leaf. In most cases, the flattened mass is closely located to the palate and sits in a slightly cupped-out depression [10, 22]. Treatment is accomplished by means of conservative surgical removal and fabrication of new dentures. While altering the denture may decrease the size of the lesion, adjustment alone will not lead to complete regression due to dense nature of the scar tissue. Recurrence is not uncommon [21].

#### 3.1.8. Epulis Granulomatosum

Epulis granulomatosum or epulis hemangiomatosis—a variant of pyogenic granuloma—presents as an overgrowth arising from a recently extracted tooth socket [23–25]. The precipitating factor in most patients is sharp specula of the alveolar bone left in the walls of the socket [1]. Clinically, it is characterized by reddish, smooth, pedunculated or sessile, nontender, rapidly growing mass with soft to firm in palpation. Surface of the lesion may be ulcerative due to secondary trauma [23, 24]. It often bleeds easily because of its high vascular content [23]. The growth may become apparent in one or two weeks after tooth extraction [1]. Lesions of larger sizes were also incidentally seen as an oral finding in patients with Klippel-Trenaunay syndrome [23]. Evaluation of socket and removal of any bony spicules or tooth fragments at the time of extraction prevent formation of an epulis granulomatosum. Excision of the raised mass and curettage of the alveolus to ensure the elimination of irritating particles is needed to treat the lesion [23–25].

#### 3.1.9. Pulp Polyp

**Figure 8:**
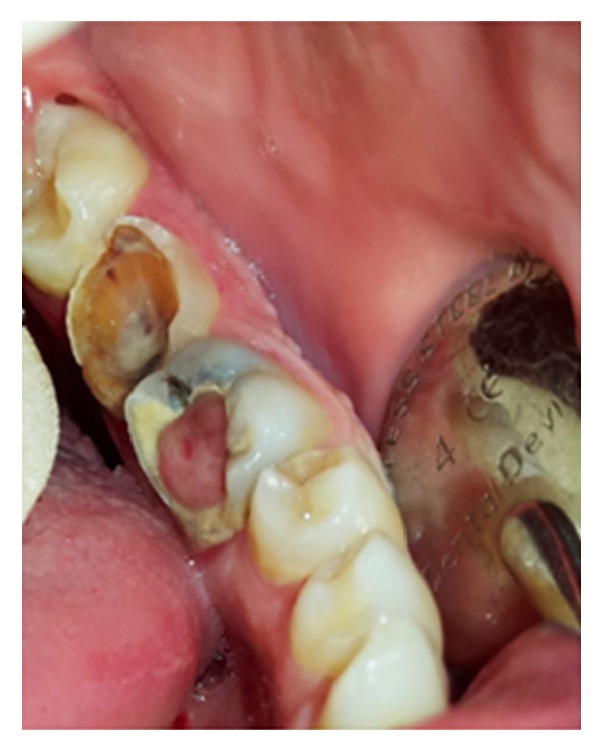
Pulp polyp associated with carious first mandibular molar with smooth surface and sessile base.

Pulp polyp or chronic hyperplastic pulpitis or pulpitis aperta is an uncommon reactive lesion, which occurs when caries have destroyed the tooth crown [1]. It appears as a smooth, soft to firm, red to pink, pedunculated, or sessile mass occupying the entire carious cavity in the affected tooth resembling an enlarged gingival tissue (Figure 8) [26, 27]. The size of the lesion varies from less than 1 cm in diameter to large masses (about 4 cm) [26, 27]. It is most frequently found in the deciduous and permanent first molars of children and young adults and is a rare phenomenon in the middle-aged adults [1, 28]. It is usually asymptomatic, but discomfort can occur during mastication. Response to electrical and thermal stimuli may be normal [28]. Periapical radiographs may show an incipient chronic apical periodontitis when pulp involvement is extensive or lingering [28]. The polyp may cover most of the remaining crown of the tooth, giving the lesion an appearance of a flashy mass [29]. A similar hyperplastic mass around a draining sinus tract of a tooth with pulpoperiapical pathology is called parulis [4]. The treatment plan of this lesion includes endodontic treatment or tooth extraction [28].

#### 3.1.10. Giant Cell Fibroma

**Figure 9:**
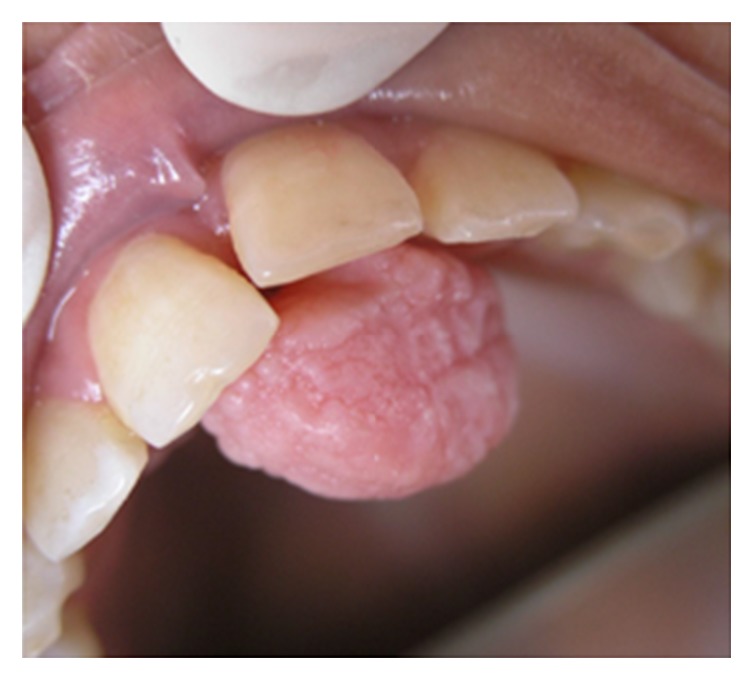
Pedunculated lesion of giant cell fibroma with granular surface on the palatal gingivae of maxillary central incisors.

Giant cell fibroma is a fibrous hyperplastic soft tissue lesion classified as an inflammatory hyperplasia [1]. However, a controversy exists about the origin and etiology of this entity [30]. It represents approximately 2% to 5% of all oral fibrous lesions and 0.4% to 1% of all oral biopsy samples [31]. Clinically, it appears as an asymptomatic, sessile, or pedunculated mass with rough surface (papillary or granular) and firm in palpation (Figure 9) [30, 31]. Despite previously mentioned reactive lesions, giant cell fibroma did not have a completely smooth surface; hence it may clinically be mistaken with lesions of squamous epithelium origin such as papilloma [10]. The color of the lesion is pink or similar to the surrounding normal mucosa and is usually less than 1 cm in diameter [10, 31]. In about 60% of cases, the lesion is diagnosed in the second to third decade of life with only 4% to 17% of giant cell fibromas being found in children younger than 10 years [31, 32]. There is a slight female predilection and gingivae are the most affected site (about 50% of cases) followed by the tongue, palate, buccal mucosa, lips, and floor of the mouth [10, 31]. Moreover, mandibular gingivae are affected twice as often as the maxillary gingivae [32]. Giant cell fibroma usually is treated by conservative surgical excision. Electrosurgery and laser therapy have been suggested as alternative modalities especially in children. Recurrence is rare [10, 28].

#### 3.1.11. Inflammatory Papillary Hyperplasia

Inflammatory papillary hyperplasia (palatal papillomatosis and denture papillomatosis) is a reactive lesion seen most often in patients with an ill-fit denture wearing all-day-long and poor oral hygiene [10, 33]. This condition is encountered in 10% to 20% of denture wearers [1, 10]. It is featured by exophytic masses with pebbly or cobblestone appearance on the hard palate beneath a denture base with or without symptoms [10]. Although these lesions appear as red, soft masses in the inflammatory stage they convert to pink and firm when they mature to fibrous stage [1]. In some cases, denture papillomatosis develops on the edentulous mandibular alveolar ridge or on the surface of an epulis fissuratum [10]. It can occur at any age. However, it is most frequently encountered in the third to fifth decade of life with a male predilection [33]. In addition to frictional irritation provoked by loose-fit dentures,* Candida albicans* has an etiologic role in this entity [1]. On rare occasions, this condition occurs on the palate of patients without denture especially in people who habitually breathe through their mouth or have a high palatal vault [10]. Less extensive lesions are resolved by removing the denture at night and improving oral hygiene. Patients can also benefit from antifungal agents. Various surgical methods have been suggested for the treatment of this lesion such as partial thickness or full thickness surgical blade excision, curettage, electro surgery, and cryosurgery [10, 34, 35].

### 3.2. Salivary Gland Lesions

A wide range of lesions arise from intraoral salivary glands, which are categorized as nonneoplastic and neoplastic entities. According to a 15-year-retrospective study by Mohan et al., 55% of lesions were nonneoplastic and 45% were neoplastic [36]. When salivary gland lesions appear as an exophytic lesion they are usually characterized by a dome-shaped or nodular mass with a smooth surface and fluctuant to firm in palpation [1]. The most common lesions of salivary gland origin are as follows (Table 2).

#### 3.2.1. Mucocele and Ranula

**Figure 10:**
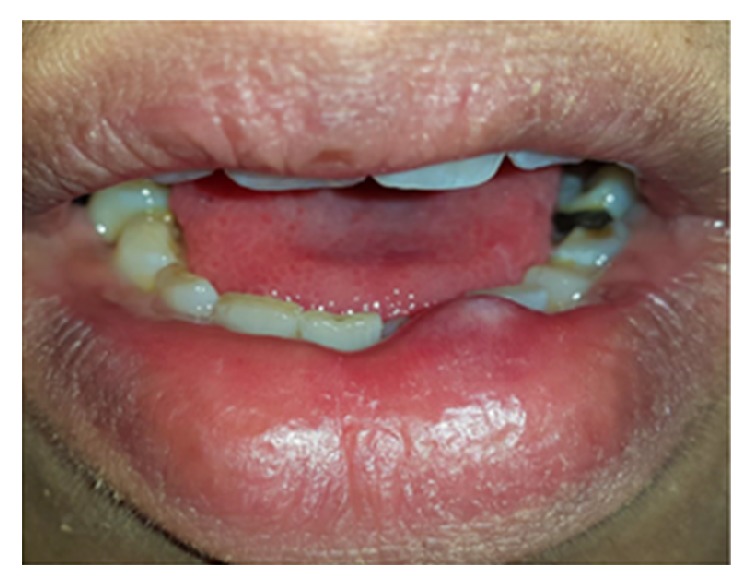
Nodular mucocele of the lower lip with smooth surface.

Mucocele is a common nonneoplastic lesion of salivary gland origin resulting from breaking or dilation of salivary ducts secondary to obstruction or local trauma [37]. Clinically, it appears as an asymptomatic, fluctuant to firm, bluish to pink, nodular, or dome-shaped mass with a smooth surface (Figure 10) [1, 37]. The size of the lesion varies from 1-2 mm to several centimeters but remains smaller than 1.5 cm in diameter [37]. The incidence is pretty high, 2.5 cases per 1000 persons with no sex predilection [38]. Lesions last from a few days to several months. Many patients mention a history of recurrent swelling with periodic rupture and release of fluid content [10, 38]. The majority of cases have been reported in the first three decades of life, and it is rare among children younger than 1 year of age [37, 38]. The lower lip is the most frequently (60%) affected site followed by buccal mucosa, anterior ventral tongue, floor of the mouth, and upper lip [10]. In some cases, mucoceles rupture and heal spontaneously; however conventional surgical treatment may be suggested in large lesions. Some alternative modalities such as cryosurgery, intralesional injection of steroids, and laser therapy have been implemented as well [37].

When a mucocele occurs in the floor of the mouth it is called ranula featuring as a bluish, dome-shaped or nodular; fluctuant exophytic lesion with a smooth surface usually located lateral to the midline [10]. Fluctuation in size considered as a pertinent feature noticed in the history of ranula. The lesion measures its smallest size early in the morning and reaches to the largest scale at the time of meals [1]. A prevalence of 0.2 cases per 1000 persons has been found, and it accounts for 6% of all oral sialocysts [39]. Most of the patients are young adults with peak frequency being in the second decade of life [39]. This lesion is treated by marsupialization or removal of the feeding sublingual gland; however marsupialization leads to recurrence in 25% of cases [1, 10].

#### 3.2.2. Pleomorphic Adenoma

**Figure 11:**
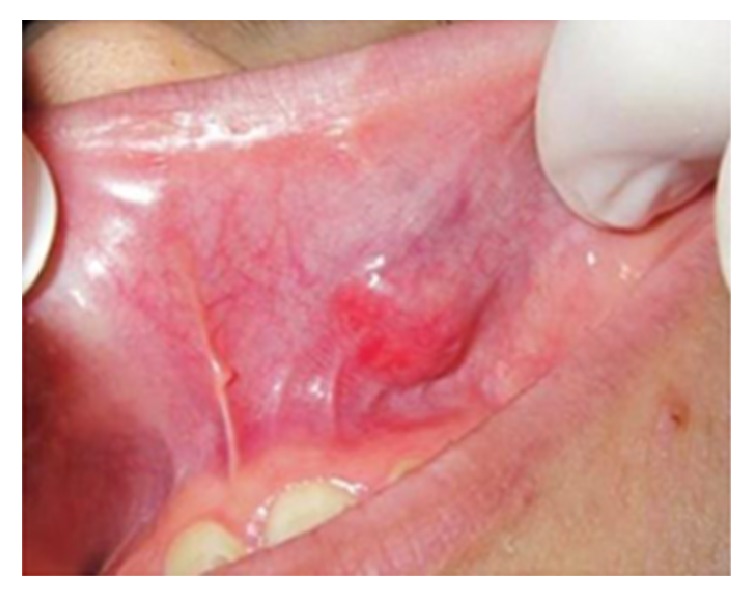
Pleomorphic adenoma involving upper lip presented as a dome-shaped exophytic lesion with smooth surface.

Pleomorphic adenoma or mixed tumor is a benign salivary gland tumor which constitutes 3% to 10% of head and neck neoplasms and about 1% of all body tumors [40, 41]. It is the most common (73%) tumor of both minor and major salivary glands [41, 42]. Corresponding to minor salivary glands, the palate is the most affected site followed by lips, buccal mucosa, tongue, floor of the mouth, pharynx, retromolar area, and the nasal cavity [41]. It is suspected when a clinician encounters a unilateral, asymptomatic, slow growing, firm, nodular, or dome-shaped mass covered by a normal-colored mucosa (Figure 11) [40, 41]. The surface of the lesion is usually intact, but ulceration of the overlying mucosa has been reported in some instances [40]. The size of the lesion varies from 1 to 5 cm with the average of 2.6 cm in diameter [41]. In large cases bone resorption has been reported [40]. It can occur at any age, but majority of patients have been reported in their fifth to sixth decades of life [41, 42]. Lip lesions tend to occur at an earlier age than that of other locations [41]. There is a female predilection with female to male ratio of 7 : 3 [41]. Malignant transformation to pleomorphic adenocarcinoma has been noticed in about 6% of cases [42]. Surgical excision usually yields promising results treated with good prognosis. A recurrence rate of 2% to 44% has been reported, and patients younger than 30 years are more likely to have relapsing lesions [41]. Risk for recurrence seems to be lower in the minor salivary gland tumors than that of major salivary glands [41].

#### 3.2.3. Mucoepidermoid Carcinoma

**Figure 12:**
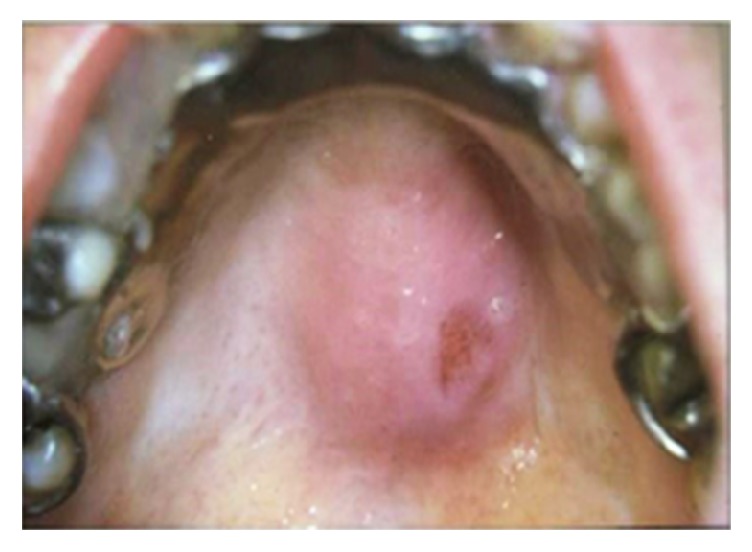
Mucoepidermoid carcinoma of the palate, presented as an exophytic lesion with smooth, ulcerated surface, and nodular base.

Mucoepidermoid carcinoma or mucoepidermoid tumor is the most common malignant salivary gland neoplasm. It accounts for about 3% of all head and neck tumors [10, 43]. Two-thirds of the lesions arise within the parotid gland and one-third within the minor salivary glands. Intraorally, palate is the most affected site followed by retromolar area, floor of the mouth, buccal mucosa, lips, and tongue. Intraosseous lesions have been also reported in some cases [10, 43, 44]. The tumor can occur at any age, but the majority of patients have been diagnosed between the third and sixth decade of life with a female predilection. Although it is rare in children, mucoepidermoid carcinoma is the most common malignant salivary gland neoplasm in childhood [10, 43–45]. Clinically, it appears as a smooth-surfaced nodular or dome-shaped, firm mass with a diameter up to several centimeters. The color of the lesion may be pink, bluish to red, or even similar to the surrounding normal mucosa (Figure 12) [1, 10, 43]. Patients are usually asymptomatic, but high grade tumors might be painful [44]. Palatal neoplasms cause bone resorption, and an ulcerative surface might be found in some tumors. [10, 44]. Low grade neoplasms are treated by surgical excision with free surgical margins. High grade ones require wide surgical excision, neck dissection, and postoperative radiotherapy. Recurrence rate is up to 60%, which mostly occurs within 1 year after treatment [45].

#### 3.2.4. Adenoid Cystic Carcinoma

Adenoid cystic carcinoma is the most common malignant tumor originated from the submandibular and minor salivary glands. It comprises 5% to 10% of all salivary gland neoplasms and 2% to 4% of all head and neck malignancies [4, 46]. It can occur in any salivary gland, but approximately 50% to 60% of cases develop within minor salivary glands. The palate is the most frequently affected site in the oral cavity. Clinically, it is characterized by a slow growing, pink to normal-colored nodular mass with a smooth surface and firm consistency [1, 46, 47]. An intraoral adenoid cystic carcinoma may exhibit mucosal ulceration, a feature that helps distinguish it from a benign pleomorphic adenoma [4]. Pain is a common finding and usually occurs early in the course of the disease before an apparent swelling develops [10]. In cases arising in the palate or maxilla, there is an evidence of bone destruction adjacent to the tumor [4, 47]. Local recurrences, perineural spreading, and distant metastases have been also reported as important features of this entity [4]. The peak prevalence of this tumor is in the fifth and sixth decades, and it is rare in people younger than 20 years [10, 46]. An almost equal sex distribution has been mentioned; however some studies showed a female predilection [10, 46, 47]. Because of the ability of this lesion to spread along the nerve sheets, radical surgical excision is suggested as the accepted treatment plan. The tumors of the minor salivary glands should be treated by local radical excision and postoperative radiotherapy. Chemotherapy is also recommended in the management of advanced and metastatic salivary gland tumors [47].

### 3.3. Mesenchymal Lesions

Peripheral oral mesenchymal tumors are considered among uncommon lesions of the oral cavity (Table 3). According to a 10-year-retrospective study by Mendez, only 4% of oral lesions were of mesenchymal origin [48]. Clinically, these lesions present as asymptomatic, slow growing, nodular, or dome-shaped masses with a smooth surface and firm consistency. These lesions are usually covered with normal mucosa unless chronically traumatized. They can be involved any site of the oral cavity [1]. The most common oral lesions with mesenchymal origin are lipoma, neurofibroma, schwannoma, lymphoma, hemangioma, and lymphangioma [49].

#### 3.3.1. Neurofibroma

**Figure 13:**
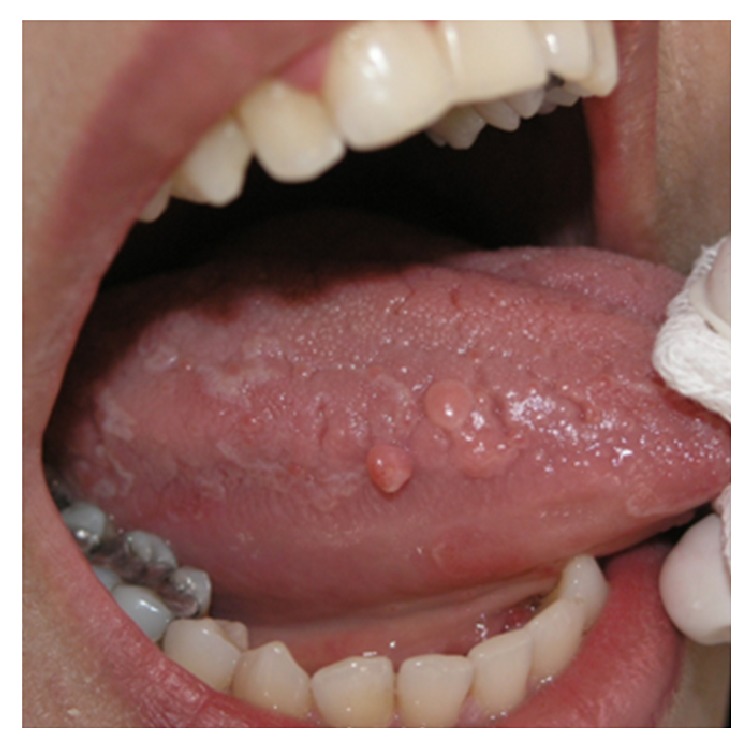
Sessile-based and smooth-surfaced exophytic lesions of neurofibroma involving dorsal and lateral border of the tongue.

Neurofibroma is the most common peripheral nerve tumor; however it is rarely seen in the oral cavity. Most cases of oral neurofibroma are multiple and as a part of neurofibromatosis syndrome, but it rarely appears as a solitary mass without visceral manifestations [10, 50]. It has been demonstrated that solitary neurofibroma is a hyperplastic hamartomatous malformation rather than a neoplastic lesion [51]. It appears as an asymptomatic, slow growing, soft to firm, nodular, or sessile mass with a smooth surface (sometimes lobulated) and pink in coloration (Figure 13) [50, 51]. It most commonly develops on the tongue followed by palate, mandibular ridge/vestibule, maxillary ridge/vestibule, buccal mucosa, lips, floor of the mouth, and gingivae with up to several centimeters in diameter [50, 51]. Intraosseous lesions have been also reported in the posterior mandible as a well-defined or poorly defined unilocular or multilocular radiolucency [51, 52]. It is most commonly observed in young adults with a peak prevalence in the third decade of life with the sex predilection being still debatable [50, 51]. The tumor is usually treated by complete excision with a low recurrence rate. However, neurofibroma may convert to neurofibrosarcoma in 5% to 15% of cases especially in multiple lesions [51].

#### 3.3.2. Schwannoma

**Figure 14:**
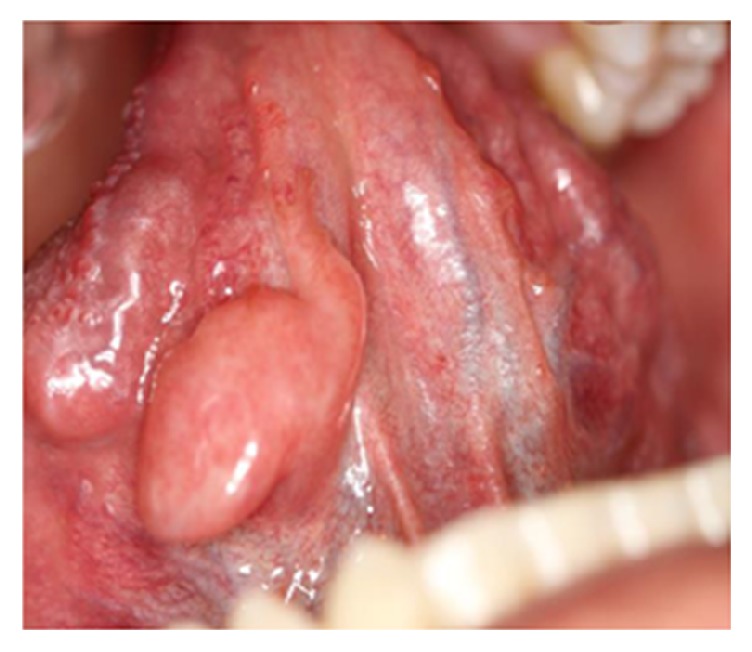
A dome-shaped schwannoma on the ventral surface of the tongue with smooth surface.

Schwannoma, also called neurilemmoma, neurinoma, or perineural fibroblastoma, is a benign tumor of neuroectodermal origin [53, 54]. Approximately 25% to 45% of lesions are seen in the head and neck with 1% being reported in the oral cavity as well [53]. Clinically, it is characterized by solitary, asymptomatic, rubbery, nodular, or sessile mass with a smooth surface and is similar to the normal mucosa in coloration (Figure 14). The size of the lesion varies from 0.5 to 4 cm in diameter [53, 54]. Intraoral lesions are frequently located in the tongue followed by palate, floor of the mouth, buccal mucosa, gingivae, lips, and vestibular mucosa [55]. Schwannoma can occur as an intraosseous lesion, which accounts for 1% of all benign primary bony tumors [54, 55]. Intrabony lesions appear as either unilocular or multilocular radiolucencies [53]. Peripheral lesions are usually painless, but tenderness may occur in some instances [10]. Pain and paresthesia are not uncommon for intrabony tumors [10]. Schwannoma has been reported in the age range of 8 to 72 years with an average age of 34 years with a slight female predilection (female to male ratio of 1.6 : 1) [53, 55]. Surgical excision is the treatment of choice and recurrence and malignant transformation are extremely rare [55].

#### 3.3.3. Lipoma

Lipoma is a benign tumor, which seldom occurs in the mouth. It constitutes 4% to 5% of all benign tumors in the body representing about 1% to 5% of all neoplasms of the oral cavity [56]. Generally, the prevalence of the lesion is balanced in both genders; however a slight male predilection has been reported. Lipoma is a rare entity in children, and most of the patients are over 40 years [57]. It presents as a slow growing, nodular, sessile, or even pedunculated mass with a smooth surface and fluctuant to soft in palpation. The superficial lesions usually show yellowish hue, while more deeply seated ones appear as a pink mass [10, 56, 57]. Lipoma varies in size from small to large masses mostly measuring less than 3 cm in diameter [10, 56]. The buccal mucosa and buccal vestibule are the most commonly affected sites, which accounts for about 50% of all cases [10]. Less affected sites include the tongue, floor of the mouth, and lips [10]. Lipoma is treated by conservative surgical excision and usually does not recur. Intramuscular lipoma has a somewhat higher recurrence rate because of problems to remove completely [4].

#### 3.3.4. Lymphoma

**Figure 15:**
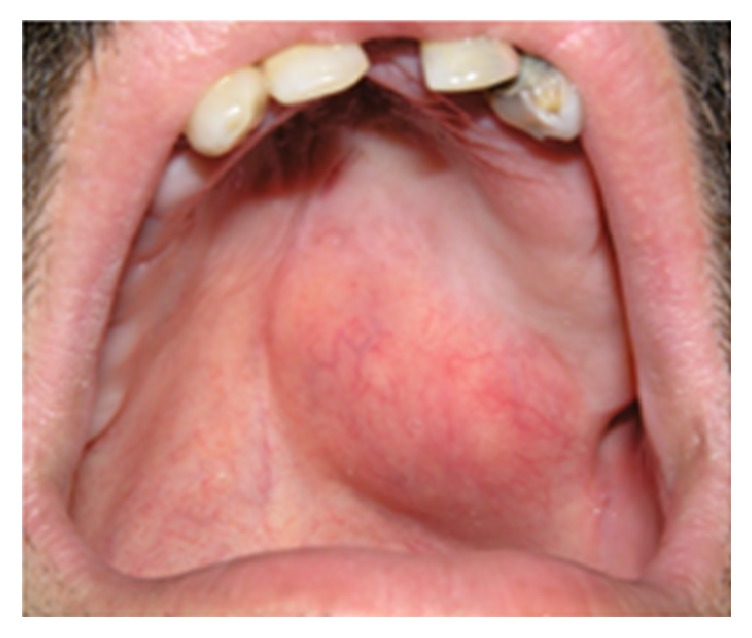
Lymphoma presented as a nodular exophytic lesion with smooth surface on the palate.

Lymphomas are heterogeneous malignant neoplasms of the lymphocyte cell lines. They can be divided as Hodgkin and non-Hodgkin lymphoma (HL and NHL). Hodgkin lymphoma rarely shows extra-nodal disease (1% of cases) unlike NHL, which arises from extra-nodal sites in 20% to 30% of patients [58, 59]. Extra-nodal lymphoma is the second most commonly encountered neoplasm after squamous cell carcinoma in the head and neck region, which accounts for 5% of all malignancies of head and neck [59–61]. Oral lymphomas usually occur secondary to a more widespread involvement through the body; however it can present as a primary lesion in the oral cavity with the prevalence of 0.1% to 2% [60]. Clinically, it appears as a nontender, smooth surface, soft to firm mass in the mouth (Figure 15) [10, 58–60]. The size of lesion varies from small to large with a pink, purplish, or normal coloration. The surface of lesion may be intact or ulcerative [10, 59, 60]. The mean age of patients with lymphoma is 56 years, and there is a male predilection [60, 61]. The most affected site for oral lesions is buccal vestibule, posterior palate, and gingivae [10, 60]. Intraosseous lesions were also reported in some cases. Bony lesions often present with low grade pain, which can mimic toothache [60]. Radiotherapy plus chemotherapy is recommended for intermediate and high grade tumors. A failure rate of 30% to 50% was demonstrated in intermediate grade lesions; however high grade lesions show a 60% mortality rate [10].

#### 3.3.5. Hemangioma

**Figure 16:**
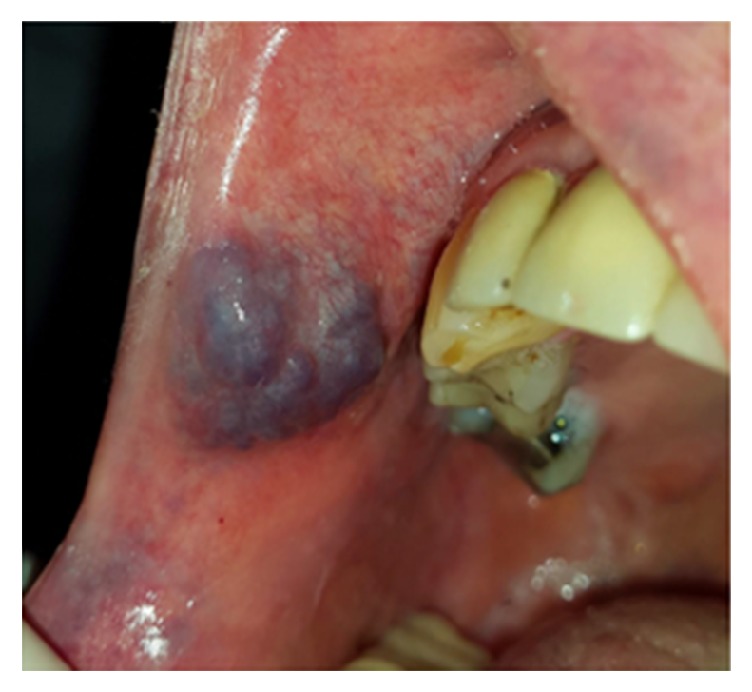
Lobulated hemangioma involving the right upper lip.

Hemangioma is a benign common tumor in the head and neck region, but relatively rare in the mouth. In the oral cavity, it can cause esthetic and functional impairment depending on its location and size [62]. The peak prevalence of this entity is described soon after birth or in early infancy; however some cases have been reported in adulthood [10, 62]. It is noted that hemangioma is the most common tumor of infancy, occurring in 5% to 10% of one-year-old children [10]. Oral lesions are most common in the lips, gingivae, tongue, and buccal mucosa [62, 63]. Eighty percent of cases occur as a single lesion, but 20% of affected patients present multiple tumors [10]. Clinically, it is characterized by an asymptomatic, soft, smooth, or lobulated, sessile mass with various sizes from a few millimeters to several centimeters (Figure 16). The color of the lesions ranges from pink to red purple, and tumor blanches on the application of pressure [63, 64]. Gingival lesions which arise from the interdental papillae can spread laterally to involve adjacent teeth [63]. There is a female predilection with female to male ratio of 3 : 1, and they usually occur in whites more often than other racial groups [10]. Treatment of hemangioma depends on its size and location. Small lesions are treated by sclerotherapy, conventional surgical excision, laser therapy, and cryotherapy. In large cases, treatment should include embolization or obliteration of the lesion and the adjacent vessels [62].

#### 3.3.6. Lymphangioma

Lymphangioma is a benign, hamartomatous tumor of lymphatic vessel origin. It has a marked tendency for the head and neck region in a way that 75% of all cases occur in this area. Almost half of the lesions are noted at birth and about 90% developed by two years of age [65]. In the oral cavity, lymphangioma mostly occurs on the dorsal surface and lateral borders of the tongue followed by gingivae, buccal mucosa, and lips [65, 66]. Pathognomonic features of tongue lymphangioma are irregular nodularity of the surface with gray to pink projections and macroglossia [66]. There is no sex predilection, and oral lesions are most common in the first decade of life [66]. The clinical manifestation of the lesion varies based on whether it is superficial or deep. Superficial lesions appear as soft exophytic masses with rough (papillary or pebbly) surface and pink to yellowish coloration. Deeper lesions are described as soft diffuse masses with normal color and smooth surface [65, 67]. Massive lesions might cause macroglossia, obstruction in upper air way, sialorrhea, and jaw deformity as well as difficulties in mastication, speech, and oral hygiene [65]. Lymphangioma is treated by surgical excision, cryotherapy, electrocautery, sclerotherapy, steroid administration, embolization, laser surgery, and radiation therapy [65].

### 3.4. Lesions with Rough Surface

This group of lesions most frequently occurs as a result of epithelial proliferation due to reaction to human papilloma virus or a neoplastic process (Table 4).

#### 3.4.1. Squamous Papilloma

**Figure 17:**
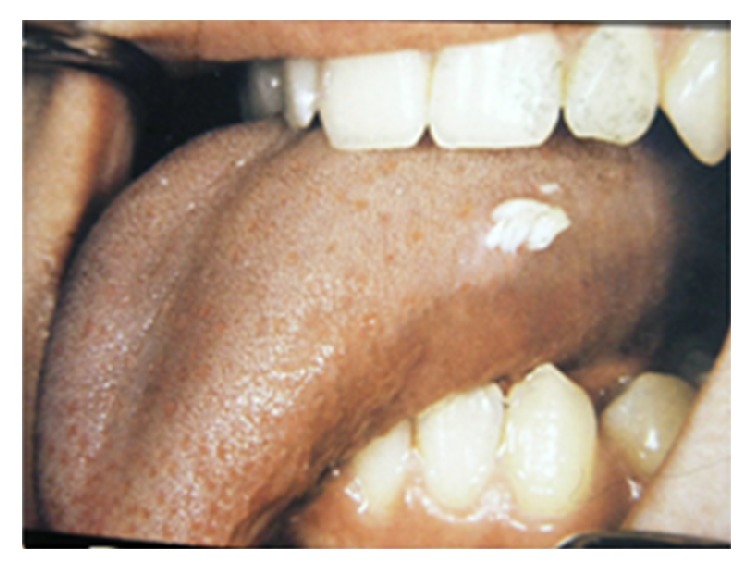
Squamous papilloma involving lateral border of the tongue with a sessile base and papillomatous surface.

Oral squamous papilloma is a benign proliferation of the stratified squamous epithelium. It occurs in one of every 250 adults and constitutes approximately 3% of all oral biopsy lesions [10, 68]. The etiologic factor is the human papillomavirus (HPV), and viral subtypes 2 and 11 have been isolated from up to 50% of oral papillomas [10]. Clinically, it appears as a single, asymptomatic, pedunculated or sessile, soft to firm mass with verrucous, granular, or papillomatous surface (Figure 17). The lesion may be white, slightly red, or normal in color [4, 68–70]. Papilloma usually enlarges rapidly to a maximum size of 5 mm with little or no change in diameter thereafter [10]. The most affected sites in the oral cavity are palatal mucosa and the tongue, but any oral surface may be involved [69]. Although papilloma can involve patients at any age it is diagnosed most often in people between 30 and 50 years [10]. Surgical excision by either scalpel or laser ablation is the treatment of choice. However, other modalities such as electro cautery, cryosurgery, and intralesional injection of interferon have been suggested [70]. Recurrence is uncommon, except for patients infected with human immunodeficiency virus [70].

#### 3.4.2. Verruca Vulgaris

Verruca vulgaris is a benign virus-induced hyperplasia of stratified squamous epithelium. It is usually encountered in children with a peak incidence between 12 and 16 years. However, about 10% of general population affect the disease in their middle age [71, 72]. Most of oral lesions are located on the vermilion border, labial mucosa, or anterior tongue [4, 10]. Clinically, it appears as an exophytic, sessile, or pedunculated lesions with papillary projections (verrucous or papillomatous) or a rough pebbly surface [10, 71]. The lesions are asymptomatic and may be pink, yellowish, or white in coloration [10]. It usually enlarges rapidly to its maximum size and remains constant for months or years thereafter [71, 72]. Approximately 23% of lesions show spontaneous regression in two months and the remaining lesions within 2 years [72]. Oral lesions are usually treated by conservative surgical excision. Meanwhile, laser therapy, cryotherapy, or electrosurgery has been also recommended. A small proportion of treated lesions show recurrence [4, 10].

#### 3.4.3. Verrucous Carcinoma

**Figure 18:**
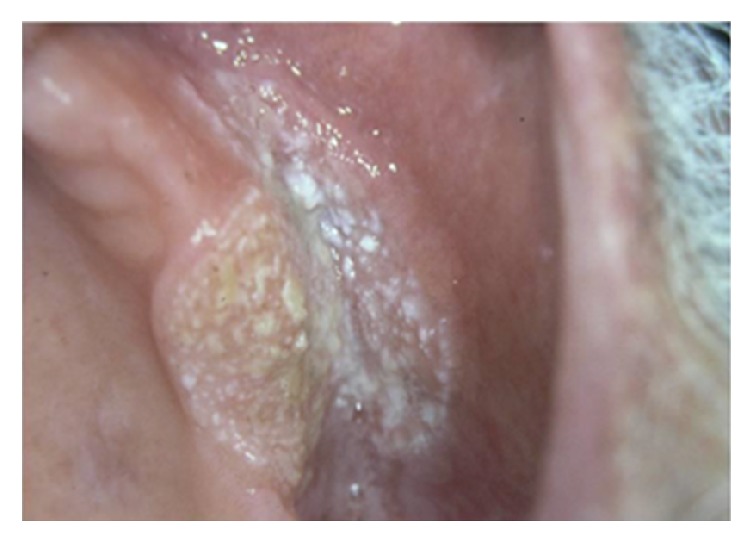
Broad-based verrucous carcinoma involving maxillary vestibular mucosa, with verrucous surface.

Verrucous carcinoma which also called Ackerman tumor, Buschke-Lowenstein tumor, florid oral papillomatosis, epithelioma cuniculatum, or snuff dipper's cancer is a nonmetastasizing low grade variant of oral squamous cell carcinoma [68, 73]. The incidence rate has been estimated as one lesion per 1.000.000 of the population each year. The etiology of the lesion is not clear, but oral lesions predominantly occur in patients habituated to areca chewing, alcohol drinking, smoking, and having poor oral hygiene. However, 15% to 51% of the lesions have been found in people without these habits [10, 68]. The most common affected sites in the oral cavity are vestibular mucosa, buccal mucosa, gingivae, and the tongue [10, 68, 73]. There is a male predilection with the majority of lesions being reported in the sixth decade [4, 68, 73]. The lesion is featured by an asymptomatic, broad-based, well-circumscribed, thick, pink to white plaque resembling a cauliflower with verruciform surface (Figure 18) [10]. Similarly simultaneous oral and genital lesions of larger sizes would suggest condyloma acuminatum [10]. Malignant transformation has been detected in 20% of the lesions [73]. Wide surgical excision, radiotherapy, and chemotherapy have been recommended for treatment.

#### 3.4.4. Squamous Cell Carcinoma

**Figure 19:**
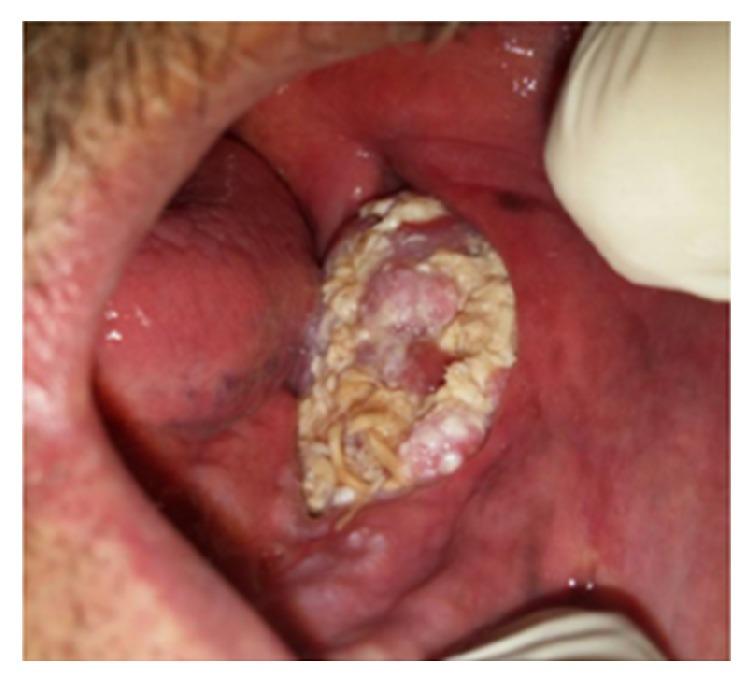
Exophytic SCC with a verrucous, necrotic, and ulcerative surface, extended from vestibule of the mandible to floor of the mouth.

Squamous cell carcinoma (SCC) accounts for 90% of all oral cancers and is considered as the eighth most frequent cancer globally [74]. Males are affected more frequently than females with a male to female ratio of 3 : 1 [10]. The median age of diagnosis is 62 years. However, the incidence of oral SCC in persons younger than 45 is increasing [75–77]. Tongue is the mostly affected site in the oral cavity followed by floor of the mouth, gingivae, palate, retromolar area, buccal, and labial mucosa [10, 75–78]. In young patients and those with congenital oral squamous cell carcinoma the tongue is the mostly affected site of involvement as well [10]. Warning signs of oral cancer include red-white lesions, ulcer lasting more than three weeks, pain of the tongue due to its mobility and sensitive nature, lump in the oral cavity or in the neck area, discomfort with speech or swallowing, mobile teeth in the absence of periodontitis, anesthesia, and earache without apparent disease. The most frequent complaints among oral cancer patients are swelling, pain, and ulceration [74]. Clinically, it can appear in various forms such as a red or white plaque, a solitary chronic ulcer or an exophytic mass [75]. Approximately 55% of the tongue lesions were exophytic-type. This type of lesions has a rough surface (verrucous) and usually is irregular in shape (Figure 19). The lesion has a broad base and it is white, pink, or red in color. Ulceration may be present in larger fungating lesions with necrotic and multicolored surface. In addition, oral SCCs are painless and firm to hard in palpation and bleeding is not an early characteristic feature [1, 10, 75]. However, in cases with destruction of the underlying bone pain may be reported, and a “moth-eaten radiolucency” with ill-defined or ragged borders is found [10]. Despite early oral cancer whose manifestations are not definitive late-stage oral SCC show quite prominent signs such as a large mass with irregular margins, ulceration, nodularity, and fixation to the surrounding tissues [74]. The treatment of intraoral SCC is guided by the clinical stage of the disease and consists of wide surgical excision, radiation therapy, or a combination of surgery and radiation therapy [10]. The recurrence rate is estimated about 33% within 2 to 96 months [79].

#### 3.4.5. Multifocal Epithelial Hyperplasia

**Figure 20:**
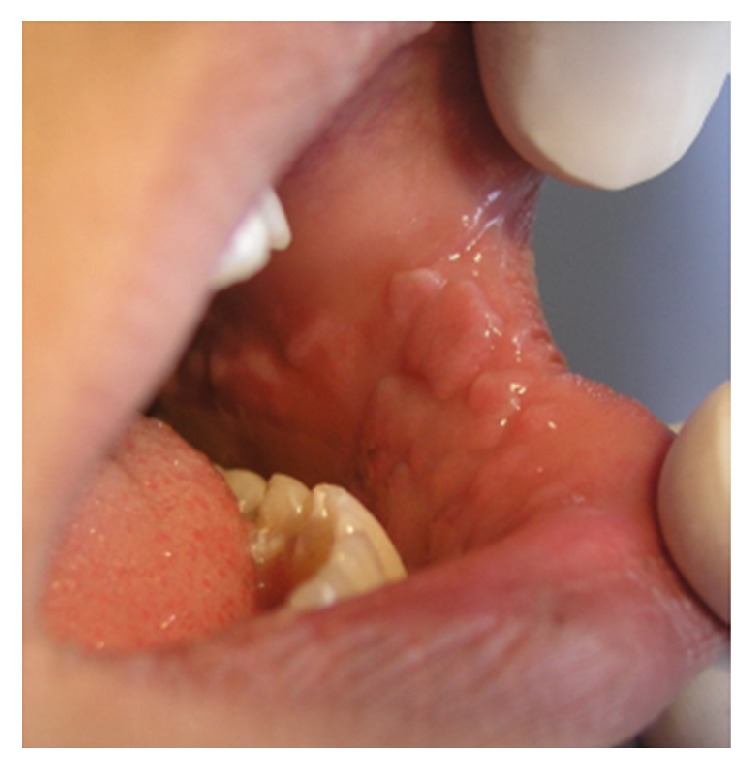
Multifocal epithelial hyperplasia presented as numerous exophytic lesions with flat surface distributed in the buccal mucosa and lower lip.

Multifocal epithelial hyperplasia, also known as focal epithelial hyperplasia, multifocal epithelial papilloma, virus epithelial hyperplasia, and Heck disease, is a virus-induced proliferation of oral squamous epithelium [10]. Although this entity is usually a childhood condition other age groups may also be affected. The frequency of the lesion ranges from 0.002 to 35% in different populations and 70% of patients are in first two decades of their life [79]. In addition, there is a female predilection with female to male ratio of 3 : 1 [79]. It appears as multiple, soft, circumscribed, nontender, flattened, or rounded papules, with either whitish color or color similar to the normal mucosa and cobblestone appearance (Figure 20) [10, 79, 80]. Most of the lesions are less than 0.5 cm, but lesions with several centimeters in size have been reported as well [10, 79]. The most frequent affected sites are labial, buccal, and lingual mucosa followed by gingivae, palate, and tonsillar mucosa [10]. There is no potential for malignant transformation [79, 80]. However, Niebrügge et al. showed malignant transformation in a female patient with long-standing Heck disease [81]. It is a self-limiting disease; hence it is recommended that only lesions located in areas subjected to trauma be excised. Risk of recurrence after therapy is minimal. There are several treatment modalities like scalpel surgery, cryosurgery, electrosurgery, and carbon dioxide laser vaporization [10, 79].

## 4. Discussion

In this review article we proposed a practical diagnostic decision tree for oral peripheral exophytic lesions as well as a brief overview about each entity. Although this group of lesions comprises a constellation of heterogeneous origins and pathogeneses, some clinical features help us categorize them to come arrive a more timely and precise differential diagnosis. We used surface texture for clinical classification of the lesions into two major groups: “lesions with smooth surface” and “lesions with rough surface.” Furthermore, the former was divided into three subgroups constituting 23 entities, and the latter constituting six lesions. While general characteristics guide to a certain group of lesions, special features point to a unique entity.

In regard to reactive hyperplastic lesions, detecting the insult factor through history taking or physical examination plays the key role for definite diagnosis.

For example, a bright red color in a soft and easily bleeding mass suggests a pyogenic granuloma, which is called a pregnancy tumor in a pregnant woman. However a peripheral giant cell granuloma presents as a more bluish, buccolingually located mass exclusively on the gingivae or alveolar ridge with a firm consistency. Meanwhile, it has a potential to cause root and bone resorption. Similarly, peripheral ossifying fibroma has a tendency to induce tooth mobility and bone destruction, but it is firm to hard in palpation with a pale pink coloration [4, 9, 10, 12, 13, 15].

Moreover, some lesions can be identified primarily by their region such as those relating to ill-fit dentures. Epulis fissuratum is seen along denture flanges as single or multiple folds, inflammatory papillary hyperplasia is located mostly on the hard palate with a pebbly or cobblestone appearance, and leaf-like fibroma presents with a narrow stalk and serrated borders beneath the base of maxillary dentures [10, 18–21].

Some exophytic lesions are found in close approximation to a tooth such as pulp polyp proliferating inside a large tooth cavity, epulis granulomatosum emanating from an extraction socket, and parulis on the alveolar mucosa or attached gingivae of a necrotic tooth [23, 25, 28].

Oral exophytic lesions of nonneoplastic salivary origin such as mucocele and ranula appear as soft and fluctuant dome-shaped lesions on oral mucosa containing salivary minor glands with a history of periodic rupture and release of fluid content. Therefore, they should not be suspected when a lesion is located on the hard palate or gingivae due to lack of submucosal salivary glands. On the other hand, a dome-shaped or nodular lesion on the posterolateral portion of the hard palate with a soft to firm consistency and slow growth rate would prompt the clinician to consider a benign salivary neoplasm such as pleomorphic adenoma in higher rankings of differential diagnosis. Moreover, accompanying pain raises the possibility of a malignant counterpart like adenoid cystic carcinoma or mucoepidermoid tumor. While a dome-shaped lesion on the lower lip is usually suggestive of a mucocele, such lesion in the upper lip should be suspected as a salivary neoplasm [37, 41, 43, 45, 46].

Peripheral oral exophytic lesions of mesenchymal origin are quite infrequent as compared with reactive or salivary lesions. Although they might show similar clinical features special characteristics such as location help differentiate them.

Neoplasms of neuronal origin mostly appear on the tongue as asymptomatic, soft to firm masses with neurofibroma being mostly multiple contrary to solitary schwannoma. In addition, concomitant eye and skin manifestations indicate neurofibromatosis. A soft fluctuant mass with a yellowish hue especially in the buccal mucosa points out a tumor of adipose tissue (lipoma). Non-Hodgkin lymphoma in the oral cavity produces a nodular pink or purplish mass with an intact or ulcerated surface on the buccal vestibule or palate with a potential for progressive growth and local destruction. Hemangioma and lymphangioma most frequently happen on the tongue sometimes with a rough surface despite their connective tissue origin. Hemangioma is characterized by a pink or purplish color and blanching on pressure, whereas lymphangioma causes macroglossia and gray to pink projections [1, 4, 10].

As a general rule, lesions originating from the epithelium present with a rough surface. An exception in this regard is focal epithelial hyperplasia with numerous small flat-end papules scattered on the oral mucosa most commonly in children and adolescence. Small finger-like projections on lesion surface are invariably seen in viral-induced lesions such as squamous papilloma, verruca vulgaris, and condyloma acuminatum. Papilloma tends to occur as a single lesion rarely exceeding 1 cm in diameter. On the other hand, verruca vulgaris and Heck disease mostly appear in multiple forms with the former being coexisted with cutaneous lesions. Coincidence of large verruca vulgaris-form lesions in the genitalia and oral cavity as well as high-risk sexual behavior refer to a condyloma acuminatum.

Squamous cell carcinoma when appears as a broad-based exophytic lesion involves the lateral border of the tongue with a rough nonhomogenous or sometimes ulcerative or necrotic surface and a progressive potential to unlimited growth. On the other hand, its nonmetastasizing counterpart—verrucous carcinoma—is mostly encountered on the vestibular or buccal mucosa in a snuff-dipper patient with a homogenous cauliflower appearance and an association with smokeless tobacco use.

## 5. Conclusion

We proposed a diagnostic decision tree regarding oral peripheral exophytic lesions divided into lesions with smooth surface and rough-surfaced lesions. Upon confronting a peripheral exophytic mass in the oral cavity a clinician should consider some features such as surface texture, shape of base, color, and consistency in order to categorize the lesion and progress along the decision tree to reach a logistic differential diagnosis.

## Figures and Tables

**Table 1:** General characteristics of smooth-surfaced oral exophytic lesions of reactive origin.

Entity	Age	Gender	Site of involvement	Surface texture	Type of base	Consistency	Color	Size	Symptom & sign	Treatment	Recurrence
Pyogenic granuloma	Children/young adult	Female	Gingivae	Smooth/lobulated	Pedunculated/sessile	Soft to firm	Shiny red/pink/purple	A few mm to several cm	Asymptomatic	Conservative surgical excision	+
Pregnancy tumor	Pregnancy period	Female	Gingivae	Smooth/lobulated	Sessile	Soft to firm	Shiny red/pink/purple	A few mm to several cm	Asymptomatic	Conservative surgical excision	+
Irritation fibroma	4–6 decades	Female	Buccal mucosa	Smooth	Pedunculated/sessile	Firm or soft	Similar to adjacent mucosa	≤1.5 cm	Asymptomatic	Conservative surgical excision	Rare
Peripheral ossifying fibroma	10–19 years old	Female	Exclusively gingivae	Smooth	Pedunculated/sessile	Firm to hard	Red to pink	<2 cm	Tooth mobility Tooth migration Bone loss	Surgical excision down to periosteum	8–20%
Peripheral giant cell granuloma	5-6 decades	Female	Exclusively edentulous ridge & gingivae	Smooth	Pedunculated/ sessile/nodular	Firm	Bluish purple	<2 cm or >2 cm	Bone loss Root resorption	Complete surgical excision	+
Epulis granulomatosum	>40 years	Female	Dental socket	Smooth	—	Soft to firm	Similar to adjacent mucosa	<1 cm to massive lesions	Asymptomatic	Conservative treatment/surgery	Not uncommon
Leaf-like fibroma	—	—	Palate	Smooth	Pedunculated/sessile	Firm	Pink	Up to several cm	Asymptomatic	Denture adjustment & surgical removal	Not uncommon
Epulis fissuratum	—	—	Maxillary alveolar ridge	Smooth/ulcerative	Pedunculated/sessile	Firm	Reddish	Up to several cm	Nontender/easily bleeding	Surgical excision & curettage	—
Pulp polyp	Children/young adults	—	Carious lesions of deciduous teeth & first permanent molars	Smooth	Pedunculated/sessile	Soft to firm	Red to pink	<1 cm to large masses	Discomfort	Root canal therapy/extraction	—
Giant cell Fibroma	3rd decade	Female	Gingivae	Rough surface	Pedunculated/sessile	Firm	Pink	<1 cm (often)	Asymptomatic	Conservative surgical excision	Rare
Inflammatory papillary hyperplasia	3–5th decades	Male	Palate	Pebbly/cobblestone	—	Soft to firm	Red to pink	Up to several cm	Asymptomatic/ symptomatic	Removal of denture at night/antifungals/ surgery	—

**Table 2:** General characteristics of smooth-surfaced oral exophytic lesions of salivary origin.

Entity	Age	Gender	Site of involvement	Surface texture	Type of base	Consistency	Color	Size	Symptom & sign	Treatment	Recurrence
Mucocele & ranula	First 3 decades of life	No sex predilection	Lower lip/floor of the mouth	Smooth	Nodular/dome shaped	Fluctuant to firm	Bluish to pink	<1.5 cm	Asymptomatic	Marsupialization	25%
Pleomorphic adenoma	5-6th decades	Female	Palate	Smooth	Nodular/dome shaped	Firm	Normal colored	1–5 cm	Asymptomatic	Surgical excision	2–44%
Mucoepidermoid carcinoma	3–6th decades	Female	Palate	Smooth	Nodular/dome shaped	Firm	Pink/bluish to red or normal colored	Up to several cm	Asymptomatic/Painful in high Grades	Wide surgical excision	Up to 60%
Adenoid cystic carcinoma	5-6th decades	Female	Palate	Smooth/ulcerative	Nodular	Firm	Pink/normal colored	Up to several cm	Pain is common/bone destruction/distant metastasis	Local radical excision + radiotherapy/ chemotherapy	—

**Table 3:** General characteristics of smooth-surfaced oral exophytic lesions of mesenchymal origin.

Entity	Age	Gender	Site of involvement	Surface texture	Type of base	Consistency	Color	Size	Symptom & sign	Treatment	Recurrence
Neurofibroma	3rd decade	No sex predilection	Tongue	Smooth	Nodular/sessile	Soft to firm	Pink	Up to several centimeters	Asymptomatic	Complete excision	Rare
Schwannoma	Average: 34 years	Female	Tongue	Smooth	Nodular/sessile	Rubbery	Same to normal mucosa	0.5–4 cm	Asymptomatic	Surgical excision	Rare
Lipoma	>40 years	Male	Buccal mucosa/vestibule	Smooth	Nodular/sessile/ pedunculated	Soft/fluctuant	Pink to yellowish	<3 cm (often)	Asymptomatic	Conservative surgical excision	High in intramuscular lesions
Lymphoma	Average: 59 years	Male	Buccal vestibule & postpalate	Smooth/ulcerative	Nodular	Soft to firm	Pink/purplish/normal	Up to several cm	Nontender painful in intraosseous lesions	Chemotherapy/ radiotherapy	—
Hemangioma	Early infancy	Female	Lips	Smooth/lobulated	Sessile	Soft	Pink to red purple	Up to several cm	Asymptomatic	Sclerotherapy/surgical excision/laser therapy/cryotherapy	—
Lymphangioma	1st decade	No sex predilection	Tongue	Smooth/pebbly	Sessile	Soft	Pink to yellowish or normal color	Up to several cm	Macroglossy/airway obstruction/sialorrhea/jaw deformity	Surgical excision/cryotherapy/ electrocautery/ steroid administration/ sclerotherapy	—

**Table 4:** General characteristics of rough-surfaced oral exophytic lesions.

Entity	Age	Gender	Site of involvement	Surface texture	Type of base	Consistency	Color	Size	Symptom & sign	Treatment	Recurrence
Squamous papilloma	30–50 years	No sex predilection	Palate & tongue	Verrucous/granular/ papillomatous	Pedunculated/sessile	Soft to firm	White/slightly red/normal	Maximum 5 mm	—	Surgical excision/electrocautery/ cryosurgery/intralesional interferon	Uncommon except for patients with HIV infection
Verruca vulgaris	12–16 years	—	Vermilion border/labial mucosa	Verrucous/ papillomatous/ Pebbly	Sessile/pedunculated	Soft to firm	Pink/yellowish/ white	Few mm	Asymptomatic	Conservative surgical excision	Low
Verrucous carcinoma	>6th decade	Male	Vestibular mucosa	Verrucous	—	Firm	White to normal	Up to several cm	Asymptomatic	Wide surgical excision/ radiotherapy/ chemotherapy	—
Squamous cell carcinoma	62 years	Male	Tongue	Verrucous	—	Firm to hard	White/pink/red	Up to several cm	Painless/moth-eaten radiolucency	Wide surgical excision/radiotherapy	33%
Multifocal epithelial hyperplasia	Childhood	Female	Labial and buccal mucosa	Cobblestone	—	Soft to firm	White to normal	Up to several cm	Nontender	Surgery/laser/ cryosurgery/ electrosurgery	Minimum
